# Study on the influencing factors of shear strength of loess mudstone interface

**DOI:** 10.1371/journal.pone.0342023

**Published:** 2026-02-03

**Authors:** Xuancheng Ren, Shijie Liu, Tao Zhu, Bowen Xu

**Affiliations:** 1 College of Geological Engineering and Geomatics, Chang’an University, Xi’an, China; 2 Shaanxi Geology Mining 908 Environmental Geology Co., Ltd., Xi’an, Shaanxi, China; 3 Shaanxi Energy Institute, Xianyang, China; Guizhou University, CHINA

## Abstract

In Loess Plateau, low shear strength of the loess–mudstone contact interface leads to the loess–mudstone landslides. However, the influencing factors of shear strength of loess-mudstone interface and the disaster-causing effect of interface landslide are not clear. Therefore, this study focus on the loess-mudstone interface, and conducts direct shear tests under different water content, density and morphology conditions. The results show that the shear strength of loess-mudstone interface is always lower than that of homogeneous loess and mudstone. An increase in moisture content leads to a continuous reduction in shear strength, whereas an increase in dry density enhances shear strength. A decrease in interface roughness also results in a reduction in shear strength parameters. Specifically, when interface roughness decreases from 1.72 to 0, cohesion decreases by 42%, and friction angle decreases by 13%. The failure modes in different interface types can be classified into three: interface shear, locked-segment, and partial locked-segment failure model. This study analyzes the influencing factors and internal mechanism of shear strength of loess-mudstone interface, reveals its landslide disaster effect, and puts forward suggestions for risk assessment of loess-mudstone landslide. The results are of great value to the potential risk assessment of loess-mudstone landslide.

## 1. Introduction

Geological environment of the Loess Plateau is highly fragile, leading to frequent soil erosion, landslides, collapses, and ground subsidence [1,2]. In recent years, large-scale hillside excavation and city-building projects on the Loess Plateau in China have resulted in the formation of numerous steep slopes [3,4]. These slopes typically exhibit a geological structure characterised by an upper loess layer overlying a mudstone [5]. The unique geotechnical composition makes such slopes highly susceptible to landslides, particularly loess–mudstone interface landslides, which are frequently triggered by rainfall and other external factors [6]. A loess–mudstone interface landslide refers to a sliding failure that occurs along the contact surface between the loess and the underlying mudstone and is one of the most common types of landslides on the Loess Plateau [7]. These landslides are inherently unstable, prone to reactivation, and often occur in clusters [8,9]. The shear strength characteristics of the loess–mudstone interface play a critical role in governing the initiation and progression of such landslides [10,11]. However, the shear strength of the loess–mudstone interface is highly influenced by rainfall, soil and rock properties, and the environmental conditions of its formation [12,13]. Therefore, investigating the shear mechanical properties of the loess–mudstone contact interface is crucial for understanding the triggering mechanisms of these landslides and for developing strategies to mitigate their severe consequences and potential risks [14].

During geological history, rock and soil masses develop numerous structural planes of varying origins through sedimentation and tectonic activities, which significantly govern their stability [15]. The loess-mudstone contact interface represents a typical example of such structural planes. The shear strength characteristics of contact interfaces are a primary indicator of their mechanical properties. In practical construction, soil tends to slip along the interface between the foundation and structures; thus, the interface is often treated as a weak plane, with low geotechnical strength parameters. The interface denotes the contact surface between two materials [16], where differences in strength and physical properties on either side of the interface may lead to failure under influences such as seismic activity, rainfall, or engineering disturbances. Therefore, the interface is commonly considered a potential failure plane. The direct shear test can simulate the shearing process of soil slipping along a “dual-layer heterogeneous” contact interface, effectively characterizing the interface’s shear mechanical properties [17,18]. Current research on interfaces has primarily investigated the shear mechanical characteristics between soil and different structural materials [19–22], such as soil–steel [23], soil–geotextiles [24–26], sand–concrete [27], sand–steel [28], and clay–concrete interfaces [29,30]. Potyondy [31] first examined the mechanical properties of various construction material–geotechnical interfaces using a strain-controlled direct shear apparatus. After that, a large number of studies showed that the strength of these interfaces was significantly lower than that of soil or structural materials. Consequently, understanding the shear mechanical properties of the loess–mudstone interface is critically important for the prevention and control of landslides.

The strength and failure modes of soil–soil and soil–structure contact interfaces are influenced by various factors, including the physical properties of the soil, moisture content, dry density, interface roughness, loading method, and normal stress [32–34]. Liu et al. [35]found that urface roughness has a significant effect on the shear behavior of concrete-soil interface. Vangla et al. [36,37] studied the impact of the sand particle morphology on sand–geotextile interfaces, concluding that the particle shape significantly affects the interface strength. Wu et al. [38] proved that factors such as soil water content, surface roughness and structural material type greatly affect the shear characteristics of soil-structure interface. Hu et al. [39] identified a critical interface roughness threshold that differentiates between low- and high-roughness failure modes, exhibiting ideal elastoplastic failure in the former and strain-localized failure with marked strain-softening and dilation in the latter. Chen [40] qualitatively studied interface roughness and found that interface strength increases with greater roughness. Ammar et al. [41] observed that enhanced drainage conditions and increased roughness at clay–hemp fiber interfaces improve sample strength. Zhu et al. [16]examined the effect of contact angle on the shear mechanics of the loess–Hipparion red clay interface, showing that shear strength varies nonlinearly with normal stress and first increases and then decreases with the interface angle. In summary, researchers have comprehensively studied soil–structure interface shear properties using laboratory tests, field monitoring, numerical simulations, and combined approaches, yielding substantial insights. However, the shear mechanical properties of contact interfaces between dual-layer heterogeneous soils remain underexplored. There are many studies on the mechanical relationship and strength of loess structure interface, but few studies on the shear behavior of loess mudstone interface [16]. In particular, the laws and causes of various factors affecting the shear properties of loess mudstone interface are still unclear, highlighting an urgent need for further investigation.

Given the high incidence and hazardous nature of loess–mudstone landslides, it is essential to conduct an in-depth investigation into the factors influencing their shear strength. Therefore, this study focuses on the loess–mudstone interface, conducting direct shear tests under varying moisture content, dry density, and interface morphology to examine their effects on interface shear strength. Based on scanning electron microscopy (SEM) and quantitative analysis of microscopic parameters, this study explores the micro-mechanisms governing the shear strength of the loess–mudstone interface under different influencing factors. By analysing these factors, the study further discusses the disaster effects of loess–mudstone landslides. The findings provide a theoretical basis for geohazard risk assessment in the Loess Plateau region.

## 2. Materials and methods

### 2.1. Sampling site

**Fig 1:**
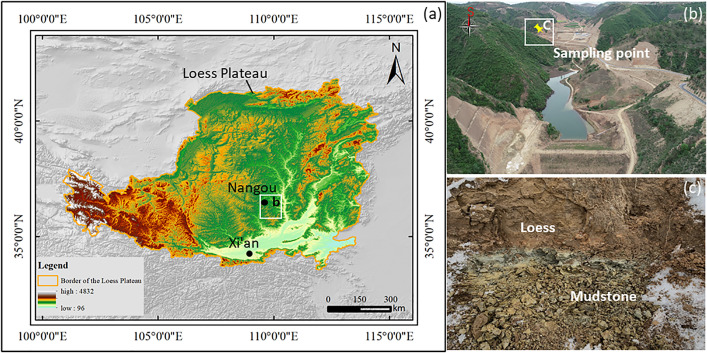
Location map of the study area and typical loess–mudstone landslides: (a) Loess Plateau; (b) loess–mudstone landslides and sampling points; (c) sampling point. (Note: The digital elevation model used in Fig 1a is derived from the ASTER GDEM (URL: https://terra.nasa.gov/data/aster-data). The boundary of loess pleatue used in Fig 1a is are available at https://doi.org/10.5281/zenodo.17948985).

The sampling site is located at the “Loess-Water Soil Geological Disaster” experimental base in Nangou, Ansai District, Yan’an City, Shaanxi Province(Fig 1a). Field investigations indicate that the cut slope at the point of loess–mudstone contact in Nangou, Yan’an exhibits various adverse geological hazards and significant damage(Fig 1b). This is attributed to the presence of natural weak planes within such slopes, which can easily become failure surfaces under the influence of external factors, such as rainfall or engineering disturbances, leading to a reduction in overall slope stability. The samples were taken from a cutting slope in Nangou, which shows a typical outcrop of loess-mudstone contact surface (Fig 1c). The contact surface is located approximately 2 m below the top of the slope, with loess samples collected from about 20 mm above the contact surface and weathered mudstone samples taken from approximately 20 mm below the contact surface.

### 2.2. Basic physical properties of loess and mudstone

**Fig 2:**
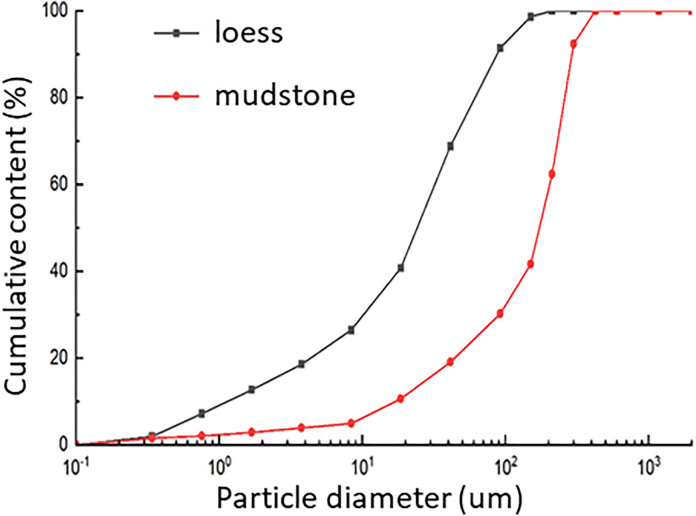
Analysis of particle size gradation of loess and mudstone for test.

Tests were conducted to determine parameters such as the particle size distribution, moisture content, specific gravity, and permeability coefficient. The moisture content was measured using the drying method. The density and dry density of loess and mudstone samples were determined using the ring knife method. The specific gravity of the loess and weathered mudstone samples was measured using a pycnometer method to ensure sample uniformity and measurement accuracy. The particle size distribution curves of the loess and mudstone were precisely determined using sieve analysis and a laser particle size analyzer (Fig 2). Proctor compaction tests were conducted to ascertain the optimum moisture content and maximum dry density of both loess and mudstone. Liquid and plastic limits were determined using a combined liquid–plastic limit apparatus, which facilitated further analysis of the soil’s plasticity and flowability. Additionally, a falling head permeability test was employed to measure the permeability coefficient. The results of physical properties test are presented in Table 1.

**Table 1:** Basic physical and mechanical indexes of loess and mudstone.

Physical properties	Loess	Mudstone
Moisture content (%)	10.31-19.80	14.00-17.19
Dry density (g/cm^3^)	1.38-1.56	1.63-1.72
Specific gravity (g/cm^3^)	2.64	2.73
Maximum dry density (g/cm^3^)	1.69	1.79
Optimum moisture content	13.86	11.72
Liquid limit (%)	30.42	38.51
Plastic limit (%)	20.92	23.19
Permeability coefficient (cm/s)	4.73 × 10^−5^	5.85 × 10^−7^
Mean grain size d_50_ (um)	24.21	172.38
d_60_ (um)	32.17	203.70
d_30_ (um)	10.20	90.80
d_10_ (um)	1.14	17.10
Coefficient of uniformity C_u_	28.22	11.91
Coefficient of curvature C_c_	2.84	2.37

By measuring the physical and mechanical parameters of loess and mudstone and comparing and analyzing them, it can be seen that the water content of loess in the study area is in the range of 10.31% − 19.80%, and the water content of mudstone is in the range of 14.00% − 17.19%. The water content of loess varies greatly in the study area, and the water content of mudstone varies little in the study area. The reason is that the loess is located on the surface of the slope, and the internal vertical joints, large pores and other structures are relatively developed, which provides a good channel for the infiltration of surface water, while the mudstone is more dense than the loess. The optimal moisture content of loess is 13.86%, and the maximum dry density is 1.69 g/cm^3^. The optimal moisture content of completely weathered mudstone is 11.72%, and the maximum dry density is 1.79g/cm^3^. The liquid and plastic limits of loess are respectively: 30.42% and 20.92%, respectively, and the plasticity index is 9.5, while the liquid and plastic limits of the lower mudstone are 38.51% and 23.19%, respectively, and the plasticity index is 15.32. It can be seen that the liquid and plastic limits of mudstone are higher than those of loess. The permeability coefficient of loess is 4.73 × 10^-5^ cm/s. The permeability coefficient of the lower mudstone is 5.85 × 10^-7^ cm/s, and the permeability coefficient of loess is much larger than that of mudstone. The compactness of the upper loess is smaller than that of the underlying mudstone, while the permeability coefficient is larger than that of the mudstone. The surface water is easy to penetrate along the dominant channel in the loess and accumulate on the surface of the mudstone, softening the contact surface of the loess mudstone. The acquisition and analysis of these physical indexes provide an important reference for the design of shear test of loess-mudstone interface.

### 2.3. Shear strength test

Direct shear tests were conducted using a strain-controlled direct shear apparatus. Based on the actual slope stress level, different normal pressure loads (100,200,300 and 400 kPa) were selected to conduct direct shear tests on the samples. The cross section of the shear box is circular, with a diameter of 61.8 mm, a cross-sectional area of 30 cm^2^ and a height of 2 cm. The loess part of the sample is located in the upper shear box, and the mudstone part is located in the lower shear box. By applying horizontal shear stress to the lower shear box, the shear of the sample at the contact between the upper and lower shear boxes, that is, the loess-mudstone contact surface, is realized. The shear displacement was recorded using a load cell (accuracy of 0.001 mm), while the deformation of the load cell ring was measured using an electrical micrometer (accuracy of 0.01 mm). The horizontal shear stress was then calculated based on the load cell coefficient. According to the ASTM D3080-04 standard, the shear test should adopt a shear rate of 0.8 mm/ min ~ 1.2 mm/ min, so all samples were sheared at a rate of 0.8 mm/min until reaching an 8 mm displacement. The peak shear stress was defined as the shear strength when the readings from the electrical micrometer showed a notable retraction. If no significant retraction occurred, the shear stress at a displacement of 4 mm was taken as the shear strength. This study focuses on the influence of different factors on the shear strength of loess-mudstone interface. The shear process of samples under all working conditions is consistent, and the influence of size effect is excluded to a certain extent.

The effective shear area gradually decreases during testing results in the actual normal stress being less than the nominal value, causing the obtained shear strength to deviate from the true strength of the soil. Therefore, correction for the effective shear area and normal stress is required so that the results more accurately reflect the mechanical characteristics of the contact interface. Firstly, the effective shear area is corrected by the single-point area correction method, and the corrected shear strength is as follows:


$$\tau^{\prime }=\frac{A_{\text{2}}}{A_{\text{0}}}\tau$$ (1)

where *A*_*0*_ is the shear area; *A*_*2*_ is the corrected actual shear area; *τ* is the shear strength of the specimen without considering area correction.

**Fig 3:**
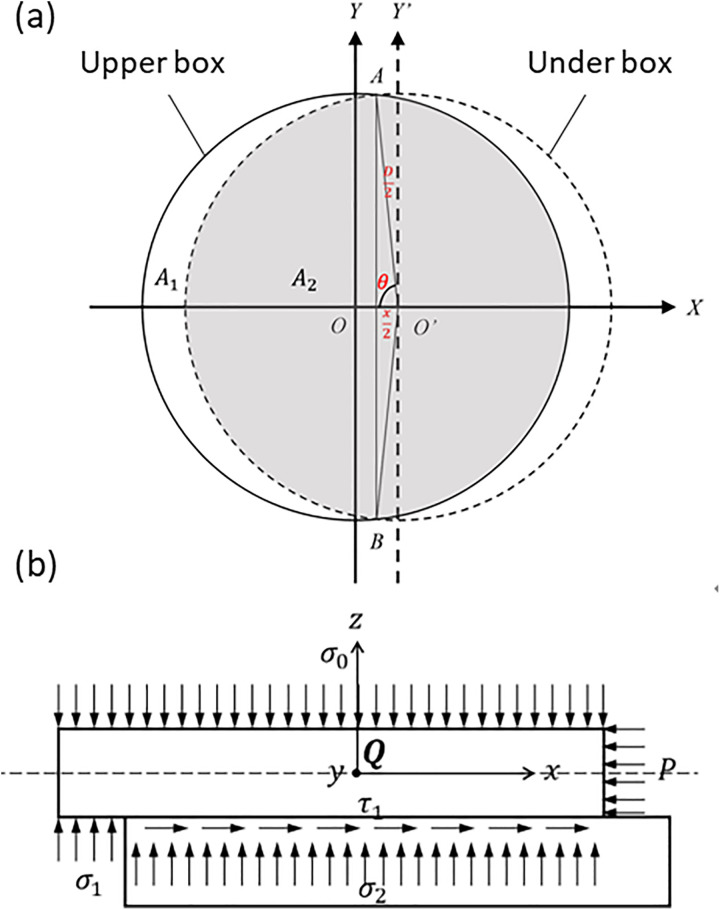
Shear strength correction. (a) Effective shear area diagram of the upper and lower shear boxes after dislocation (the gray part is the actual shear area after dislocation); (b) force diagram of the soil sample in the upper shear box after shear dislocation.

Then the effective normal stress is corrected, and the corrected normal stress on the effective shear plane is:


$$\sigma^{\prime }=\sigma_{\text{0}}-\frac{\tau^{\prime }l}{s\pi }[\pi -\text{2}\alpha +\text{sin}(\text{2}\alpha )]$$ (2)

where *σ*_*0*_ is the uncorrected normal stress; *τ’* is the corrected shear strength; *s* is the shear displacement; *α* is the central angle in Fig 3; *l* is the height of the upper the shear box.

### 2.4. Experimental conditions and sample preparation

This study used multiple experimental conditions (Table 2) to investigate the effects of various moisture contents, dry densities, and contact interface morphologies on the shear strength of the loess–mudstone interface. A total of 11 test groups were designed, each group contained 4 samples, and the normal stresses were set to 100,200,300 and 400 kPa, respectively. Each sample was set up three repeated experiments to ensure the reliability of the experimental data. Considering that rainfall and engineering disturbances primarily affect the loess layer, only the moisture content and dry density of the upper loess layer were adjusted during the tests, while the moisture content and dry density of the lower mudstone remained constant. Based on the natural parameter ranges of the loess in the study area, four moisture contents (10%, 13%, 16%, and 19%) and four dry densities (1.4, 1.45, 1.5, and 1.55 g/cm^3^) were selected. All samples were fully consolidated by jacks with preset normal pressure during sample preparation, and the consolidation state of undisturbed loess and mudstone was simulated as much as possible.

**Table 2:** Design of direct shear test conditions.

		Moisture content (%)		Dry density (g·cm^-3^)		
Group No.	Interface morphology	loess	mudstone	loess	mudstone	Normal pressure (kPa)
1	smooth interface	10	15	1.5	1.7	100, 200, 300, 400
2	smooth interface	13	15	1.5	1.7	100, 200, 300, 400
3	smooth interface	16	15	1.5	1.7	100, 200, 300, 400
4	smooth interface	19	15	1.5	1.7	100, 200, 300, 400
5	smooth interface	13	15	1.4	1.7	100, 200, 300, 400
6	smooth interface	13	15	1.45	1.7	100, 200, 300, 400
7	smooth interface	13	15	1.55	1.7	100, 200, 300, 400
8	smooth interface	13	15	1.5	1.7	100, 200, 300, 400
9	stepped interface	13	15	1.5	1.7	100, 200, 300, 400
10	shallow serrated interface	13	15	1.5	1.7	100, 200, 300, 400
11	deep serrated interface	13	15	1.5	1.7	100, 200, 300, 400

**Fig 4:**
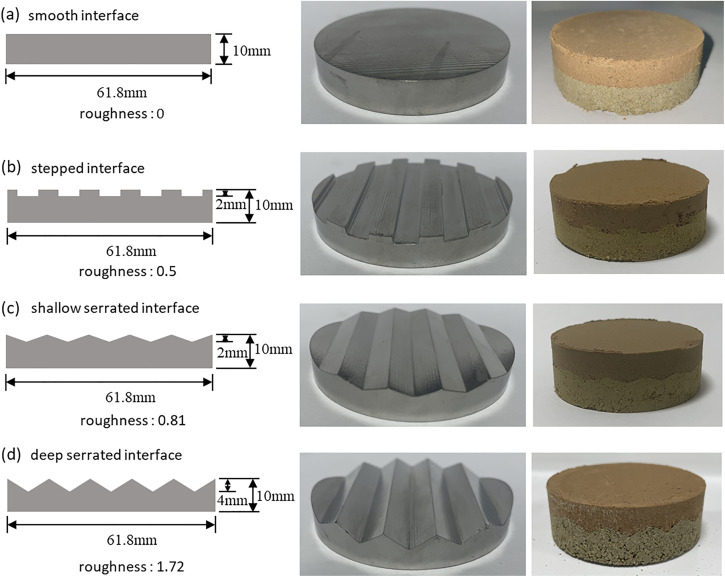
Direct shear specimens with different interface morphologies. (a) smooth interface; (b) stepped interface; (c) shallow serrated interface; (d) deep serrated interface.

The shear deformation modes of structural interface in geotechnical mechanics can be divided into three basic types: climbing, climbing gnawing and gnawing. With the increase of sawtooth fluctuation angle and normal stress, the shear deformation modes of structural interface gradually evolve from climbing, climbing gnawing to gnawing [41]. Many studies have shown that the peak-valley distance of the undulating area of the sample mold accounts for 20% −40% of the height of the entire mold [42,43]. For the sample preparation mold with a height of 10 mm in this study, the peak-valley distance range is between 2 mm and 4 mm. Therefore, this study realizes the simulation of different interface types by changing the undulating angle. In order to more accurately analyze the influence of interface morphology changes on the shear mechanical properties and failure modes of the interface, two extreme interface forms of undulating angle 0 ° (smooth type) and undulating angle 90 ° (step type) were selected, and the peak-valley distance was selected as 2 mm (i.e., the shallow serrated interface with an undulating angle of 18 °) and 4 mm (i.e., the deep serrated interface with an undulating angle of 33 °) as the transition of the extreme state(Fig 4). To prevent displacement of the contact surface during shearing due to sample compression, the compression degrees of loess and mudstone under different normal stresses were first determined through consolidation tests, allowing for the calculation of the actual density, which was then used to prepare the contact interface samples. During preparing the sample, the lower mudstone sample is first filled into the ring knife and put into the sample preparation mold (Fig 4). After flattening with a jack, the mold on the upper layer of the sample is taken out and filled into the loess sample for pressure and leveling again. The loess-mudstone interface sample that meets the design size can be obtained to meet the repeatability and error control of sample preparation.

To quantify the impact of changes in contact interface morphology on the shear strength of the loess–mudstone interface, interface roughness was employed as a parameter to represent different interface morphologies [44]. The conventional sand filling method is based on the physical relationship between surface area and volume to calculate the degree of concavity and convexity of the contact surface. However, in the study of the mechanical behavior of the contact surface, the most direct reflection of the influence of roughness is the area affected by the undulating area, and the shear failure of the contact surface only occurs within a specific depth. The standard sand volume filled within this depth range is the effective sand filling volume. Therefore, the influence area and shear influence range of convex body fluctuation are considered in the determination of contact surface roughness. The roughness of the interface of different forms used in this paper was measured by the modified sand filling method. The results are as follows: deep serrated (1.72)> shallow serrated (0.81)> stepped (0.5)> smooth (0).

### 2.5. SEM test

Scanning electron microscopy (SEM) analysis is a key technology to study the microstructure of rock and soil, especially to observe its pore structure and particle morphology [45]. SEM tests were conducted using the *FEI Quanta 400 FEG* environmental scanning electron microscope. The PCAS image processing software is used to binarize the microscopic image to extract the microstructure parameters of the contact interface, and then reveal the variation law of the microscopic elements of the contact surface under different working conditions, and analyze the microscopic mechanism of the change of the interface strength from a quantitative point of view. Avoid the influence of subjective factors in SEM analysis [46]. In this study, scanning electron microscopy and microscopic parameter analysis were carried out on the loess-mudstone interface of 132 shear specimens after direct shear tests under different working conditions to ensure the reliability of the experimental results.

Firstly, the image is binarized according to the set gray threshold T to distinguish soil particles and pores. In order to ensure accurate identification, the threshold is gradually adjusted until a clear distinction between particles and pores is achieved. Then, parameters such as pore throat closure radius and minimum pore area are set to filter out small connected parts and too small areas, so as to extract the real pore structure. Finally, the system outputs analysis parameters and statistical data to accurately quantify the microstructure characteristics. Four microstructure parameters, porosity, shape coefficient, fractal dimension and probability entropy, are calculated. The porosity is the ratio of the pore area to the total image area. The larger the value is, the larger the pore area is. The shape coefficient characterizes the complexity of the geometric boundary of the block, and characterizes the roundness and roughness of the block, which can be calculated by the area and perimeter of the block. The fractal dimension describes the self-similarity of the block and the contour, and reflects the change rate of the area and length under different measurement scales. The larger the fractal dimension is, the more complex the pore structure is, which can be calculated by the pore perimeter and pore area. The probability entropy reflects the overall arrangement of pores and particles. The smaller the value is, the higher the orderliness is. It can be obtained by the number of locations in the range of pore orientation angle, the number of pores in each location and the total number of pores.

## 3. Results

Based on the determination of physical and mechanical properties of loess and mudstone under natural conditions, this study investigated the shear strength variations of loess-mudstone interfaces under multiple influencing factors, including moisture content, dry density, and interface morphology, through direct shear tests. Scanning electron microscopy (SEM) tests were performed on shear failure surfaces of specimens under different influencing conditions to provide a microstructural characterization of the shear failure process at loess-mudstone interfaces.

### 3.1. Moisture content

**Fig 5:**
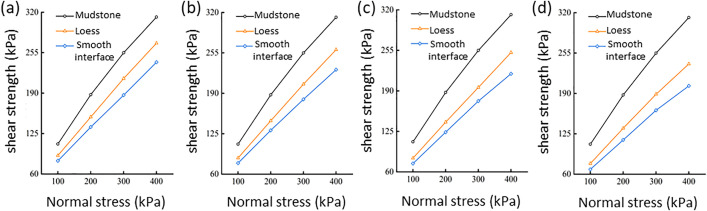
Relationship between shear strength and normal stress for loess and the smooth interface under different moisture content conditions: loess at (a) 10%, (b) 13%, (c) 16%, and (d) 19% moisture contents.

The shear stress–shear displacement relationships were obtained for pure loess under various moisture contents, the loess–mudstone contact interface under different upper loess moisture contents, and pure mudstone (S1 Fig). Both pure loess and pure mudstone exhibited significant strain-hardening characteristics under different normal pressure conditions, while the loess–mudstone contact interface showed a degree of strain softening. Under identical normal stress conditions, the shear strength of the loess–mudstone contact interface samples was consistently the lowest across all moisture conditions, followed by the loess samples, while the mudstone samples exhibited the highest shear strength (Fig 5). This indicates that the contact surface is a zone of weakness. As the moisture content of the loess increased from 10% to 19%, its shear strength declined linearly, with a relatively uniform rate of decrease. Compared to loess, the moisture content has a more pronounced effect on the shear strength of the loess–mudstone smooth contact interface. When the moisture content of the upper loess rose from 10% to 19%, the shear strength of the loess–mudstone contact interface decreased significantly under the same normal stress conditions, with the impact of moisture becoming more pronounced at higher normal stresses.

**Fig 6:**
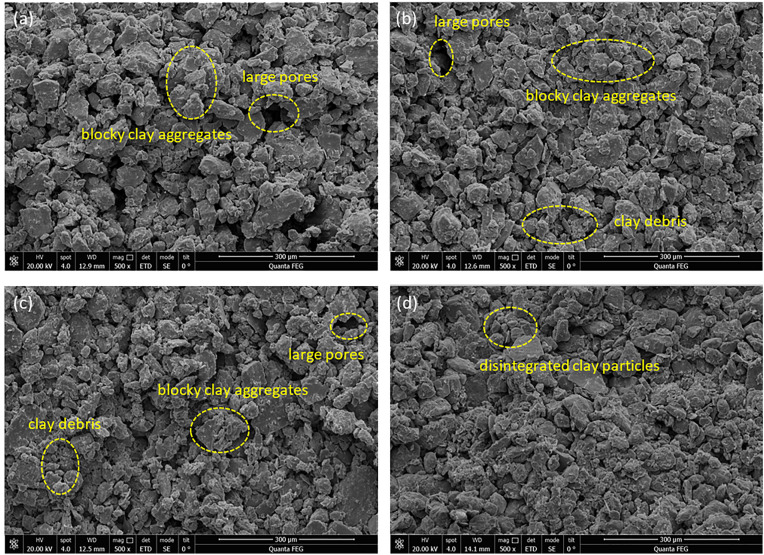
Microstructural scanning images of contact surface under different moisture contents of upper loess: (a) w = 10%, (b) w = 13%, (c) w = 16%, (d) w = 19%.

Moisture contents of the upper loess also changes the microstructure of the interface after shear failure (Fig 6). At lower moisture contents, the soil particles are larger and loosely arranged, with numerous voids present. Small clay aggregates fill the spaces between larger particles, creating internal bonding (Fig 6a). In this state, the shear failure of the contact surface primarily manifests as mechanical friction. As the moisture content increases, water accumulates at the contact surface, leading to an increase in debris and clay particles. Some larger particles become bonded by the clay, causing the pore area to gradually decrease (Fig 6b, c). When the moisture content reached 19%, the bonding materials within the clay aggregates softened and disintegrated into finer clay particles, filling the voids and resulting in the disappearance of larger pores (Fig 6d). The disintegrated clay particles, due to the adsorption of thick water films, act as lubricants, reducing the shear strength of the contact surface.

### 3.2. Dry density

The relationships between shear stress and shear displacement were obtained for pure loess, loess–mudstone contact interfaces under different upper loess dry densities, and pure mudstone control samples (S2 Fig). Pure loess and mudstone samples typically exhibited strain-hardening characteristics; thus, the shear stress at a displacement of 4 mm was used as the shear strength for these conditions. Conversely, the loess–mudstone contact interface samples demonstrated a degree of strain softening, with the peak shear strength (prior to any significant decline) taken as the shear strength.

**Fig 7:**
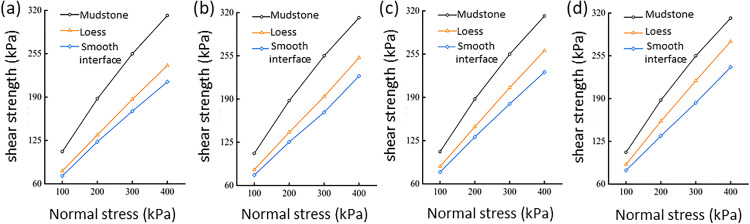
Relationship between shear strength and normal stress for loess and the smooth interface under different dry density conditions: loess with dry densities of (a) 1.4 g/cm^3^, (b) 1.45 g/cm^3^, (c) 1.5 g/cm^3^, and (d) 1.55 g/cm^3^.

Under identical normal stress conditions, the shear strength of the loess–mudstone smooth contact interface samples consistently remained the lowest (Fig 7), indicating that this contact surface is a zone of weakness within the loess–mudstone dual-layer heterogeneous structure. As the dry density increased from 1.4 to 1.55 g/cm^3^, the shear strength of the loess exhibited a continuous increase, following a linear relationship. The increase in shear strength for the loess–mudstone smooth contact interface was relatively minor. At a normal stress of 400 kPa, when the dry density of loess increased from 1.4 g/cm^3^ to 1.45, 1.5, and 1.55 g/cm^3^, the shear strength of the loess increased to 12.22, 21.80, and 33.85 kPa, respectively. In comparison, the shear strength of the contact interface increased by 7.20, 11.66, and 18.09 kPa, respectively.

**Fig 8:**
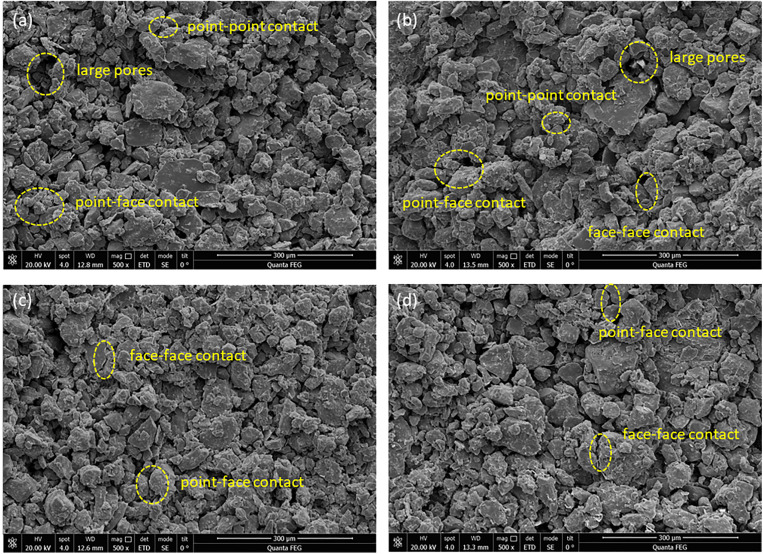
Microstructural scanning images of the contact interface under different dry densities of the upper loess: (a) ρ_d_ = 1.4 g/cm^3^, (b) ρ_d_ = 1.45 g/cm^3^, (c) ρ_d_ = 1.5 g/cm^3^, (d) ρ_d_ = 1.55 g/cm^3^.

As the dry density of the upper loess increases, significant changes occur in the microscopic features of the contact surface’s pores (Fig 8a-d), including a reduction in both the overall number of pores and the average pore area, resulting in decreased porosity. This is attributed to the increased dry density, which causes more soil particles to embed and adhere to the contact surface, filling the existing pore structure. Consequently, larger pores gradually transform into smaller and micro-pores, and the arrangement of particles shifts from loose to dense, enhancing the interlocking effect [47].

### 3.3. Interface morphology

**Fig 9:**
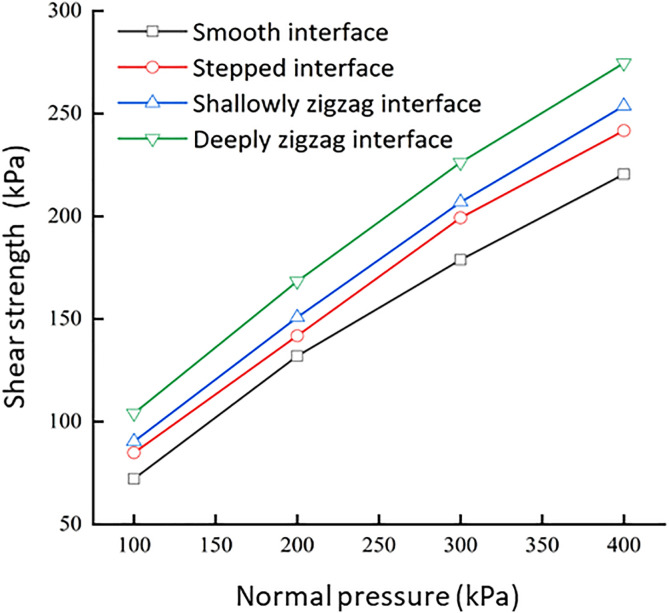
The relationship between shear strength and normal stress under different interface morphology.

The morphology of the contact interface has a significant impact on the shear strength of the samples (S3 Fig). The shear strength of the loess–mudstone contact interface increased with an increasing normal stress. Under identical normal stress conditions, the deep serrated contact interface exhibited the highest shear strength, followed by the shallow serrated, stepped, and, finally, the smooth contact interface (Fig 9). At normal pressures of 100, 200, 300, and 400 kPa, the differences in shear strength between the deep serrated and smooth contact interfaces reached 31.86, 36.38, 47.41, and 54.17 kPa, respectively. Under identical normal stress conditions, the shear strength decreased in the following order: deep serrated > shallow serrated > stepped > smooth. However, as normal pressure increased, the influence of interface roughness gradually diminished.

**Fig 10:**
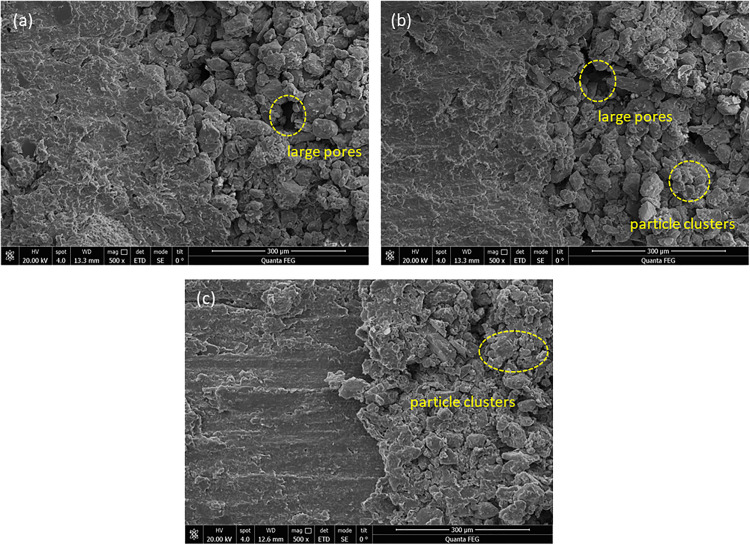
Microstructural scanning images of shear sections with different contact surface morphologies: (a) stepped, (b) shallow serrated, and (c) deep serrated.

The microstructure of the sample after shear failure is also affected by the interface morphology (Fig 10). The results indicate that for the stepped contact interface, the loess particles are larger and more porous, while the mudstone appears relatively loose, with no significant signs of shear sliding observable (Fig 10a). In contrast, the deep serrated contact interface exhibits smaller loess particles and pores, with the mudstone being denser and showing clear evidence of shear sliding (Fig 10c). The shallow serrated contact interface displays pore characteristics that fall between the two, also exhibiting signs of shear sliding (Fig 10b). During the shearing process, clusters of loess particles disintegrate into smaller fragments that fill the existing larger voids. The stepped contact interface, characterized by larger pores and lower fill levels, has loosely connected particles and a low interlocking degree, resulting in a reduced force required for shear failure, with only minor shear traces observable on the mudstone surface. Conversely, the deep serrated contact interface, with its smaller pores, high fill density, and tightly interlocked particles, has a greater force to overcome during shear, resulting in more pronounced shear traces.

## 4. Discussion

The macroscopic mechanical properties of geotechnical materials are intrinsically linked to their microstructural characteristics. To investigate the shear failure process and strength evolution at loess-mudstone interfaces, this section discusses the variations in shear strength parameters and microstructural parameters under different conditions, including moisture content of the overlying loess, dry density, and interface morphology. The macroscopic shear failure surface characteristics across diverse interface morphologies are systematically analyzed, aiming to elucidate the mechanisms governing strength variations at loess-mudstone interfaces.

### 4.1 Moisture content and dry density effects

**Fig 11:**
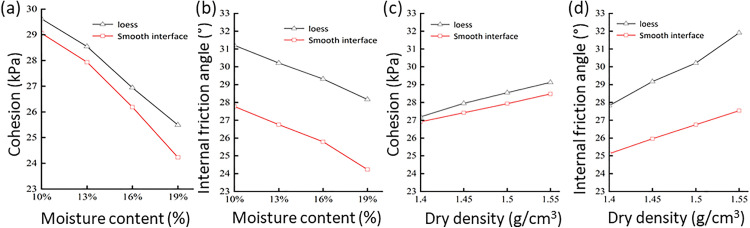
Shear strength parameters of loess and smooth interface under different water content and dry density conditions: (a) Cohesion under different moisture content conditions. (b) Internal friction angle under different moisture content conditions. (c) Cohesion under different dry density conditions. (d) Internal friction angle under different dry density conditions.

The shear stress–shear displacement curves from the direct shear tests allowed for the determination of the shear strength for loess and loess–mudstone smooth contact interfaces under different moisture contents. Linear fitting was subsequently performed to identify the trends of cohesion and internal friction angle with various moisture contents. As shown in Fig 11(a) and (b), the cohesion and internal friction angle of loess consistently remained higher than those of the loess–mudstone smooth contact interface across different moisture contents. Both loess and the loess–mudstone smooth contact interface exhibited an overall decreasing trend in cohesion with an increasing moisture content [9]. Specifically, when the moisture content of loess increased from 10% to 19%, its cohesion decreased by 3.76 kPa (approximately 13%), while the cohesion of the loess–mudstone smooth contact interface declined by 4.65 kPa (19%), indicating a more significant impact of moisture content on the cohesion of the contact surface [48]. Notably, when the moisture content of the loess rose from 10% to 13%, only a slight decrease in cohesion was observed; however, further increases in moisture content led to a pronounced decline in cohesion. This suggests the existence of an optimal bonding state for the loess between moisture contents of 10% and 13%.

The effects of dry density on the cohesion and internal friction angle of both materials were observed (Fig 11 c, d). Within the tested range of dry densities, the cohesion and internal friction angle of loess consistently remained higher than those of the loess–mudstone smooth contact interface. As dry density increased, the cohesion of both loess and the contact interface generally exhibited an upward trend [49]. When the dry density of loess increased from 1.4 to 1.55 g/cm^3^, the cohesion approximately increased 9%, similar with the contact interface. With the increase in dry density, the friction angles of both loess and the contact interface also increased. Specifically, when the dry density of loess increased from 1.4 to 1.55 g/cm^3^, the internal friction angle of loess rose by 3.44° (approximately 13%), while the internal friction angle of the contact interface increased by 1.86° (approximately 7%), indicating that the effect of dry density on the internal friction angle of the contact interface is relatively small [50].

**Fig 12:**
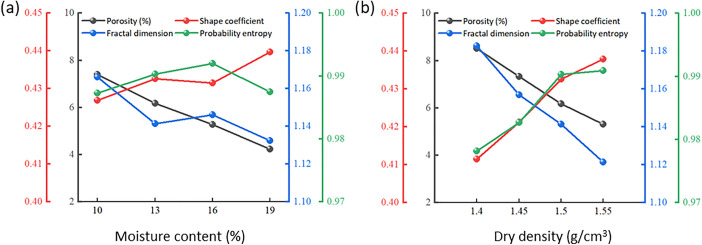
The variation law of microstructure parameters of contact interface under different water content and dry density conditions: (a) Moisture content; (b) Dry density.

The SEM images of the contact surface under different water contents were processed by PCAS to obtain the variation of the microstructure parameters [51]. As the moisture content of the upper loess increases, the porosity of the contact surface consistently decreases (Fig 12a). This reduction is attributed to the softening of the soil at the contact surface due to an increased moisture content, which weakens the bonding between particles [52]. Consequently, under a normal pressure and shear force, more particles are compressed and fill the pore spaces, leading to lower porosity. Additionally, as the moisture content increases, the pore structure of the contact surface becomes increasingly compacted, with poorly developed pore structures and an increase in small and micro-pores. The shapes of these pores tend to become more rounded, resulting in a larger average shape coefficient and a gradual decrease in the average fractal dimension. The pore probability entropy and directional fractal dimension did not exhibit significant trends.

Additionally, the average shape coefficient of the pores increases with an increasing dry density (Fig 12b). The rise in dry density leads to a reduction in the number of large pores, an increase in the number of small and micro-pores, and a decrease in the variation between the long and short axes of small pores, resulting in a more rounded pore structure that exhibits greater stability and isotropy [53]. In contrast, the average fractal dimension decreases as dry density increases. The wear and fragmentation of particles, along with the gradual filling of pores, contribute to a less developed and more dispersed pore structure, reducing the structural adjustment of the contact surface under shear forces and enhancing overall stability. Moreover, the pore probability entropy slightly increases with a rising dry density, while the directional fractal dimension does not exhibit significant patterns. This lack of a discernible trend is attributed to the increased dry density causing more soil particles to randomly embed or adhere to the contact surface, leading to a more random arrangement of pores and a more uniform distribution, without clear directional characteristics.

**Fig 13:**
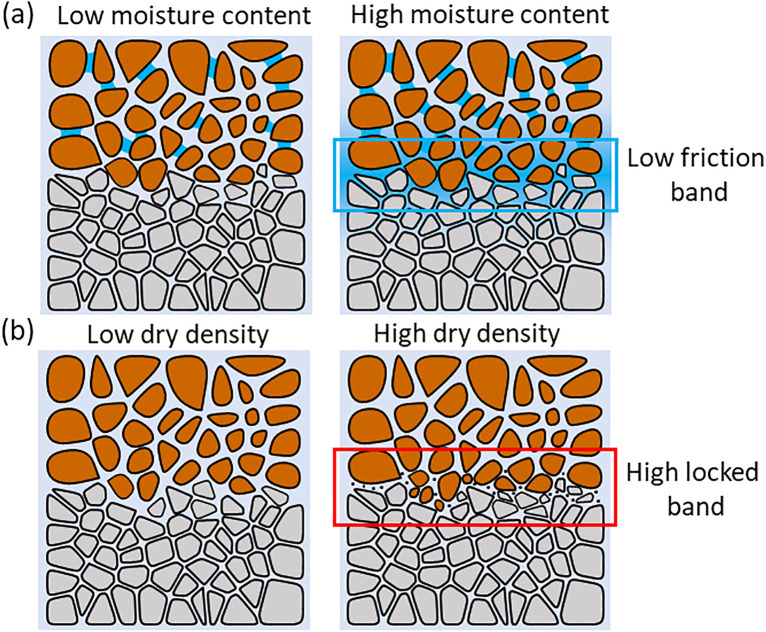
The microstructure diagram of the sample under different water content and dry density conditions: (a) Moisture content; (b) Dry density.

As the moisture content of the upper loess increases, more water accumulates at the contact surface [54]. This results in the softening and disintegration of the cementing materials within the clay aggregates, breaking them down into finer clay particles that subsequently fill the pores. The disintegrated clay particles adsorb a thick bound water film, forming a low-friction zone (Fig 13a). This low-friction zone facilitates the relative displacement of soil particles by acting as a lubricant [6], thereby reducing the shear strength of the contact surface.

As the dry density of the upper loess increases, a greater number of soil particles become embedded or adhered to the contact surface, filling the original pore structure of the interface (Fig 13b). In particular, the arrangement of loess and mudstone particles transitions from a loosely suspended structure to a more compact configuration, enhancing the locking effect. This strengthening of interparticle locking results in a increase in the macroscopic shear strength of the contact surface [47].

### 4.2. Shear failure modes of different interface morphology

**Fig 14:**
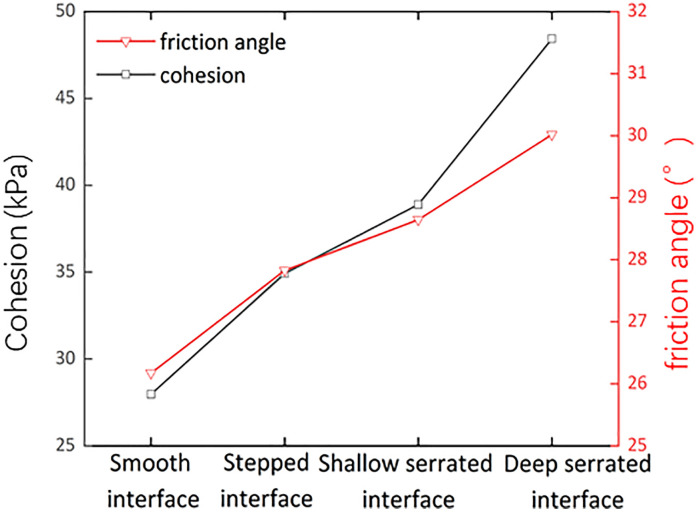
The change of shear strength parameters under different contact interface morphology.

The morphology of the contact interface has a pronounced effect on both cohesion and the internal friction angle [55]. In this study, the cohesion and internal friction angles for the four designed contact morphologies were ranked as follows: deep serrated > shallow serrated > stepped > smooth (Fig 14). When the contact interface changed from smooth to deep serrated, the roughness increased from 0 to 1.72, resulting in a cohesion increase of 20.47 kPa and an internal friction angle increase of 3.84°. Compared to the smooth contact interface, these changes represented enhancements of 73% and 15% for cohesion and the internal friction angle, respectively. The substantial increase in cohesion relative to the internal friction angle indicates that altering the contact interface morphology primarily enhances shear strength by influencing the cohesion of the interface [56].

**Fig 15:**
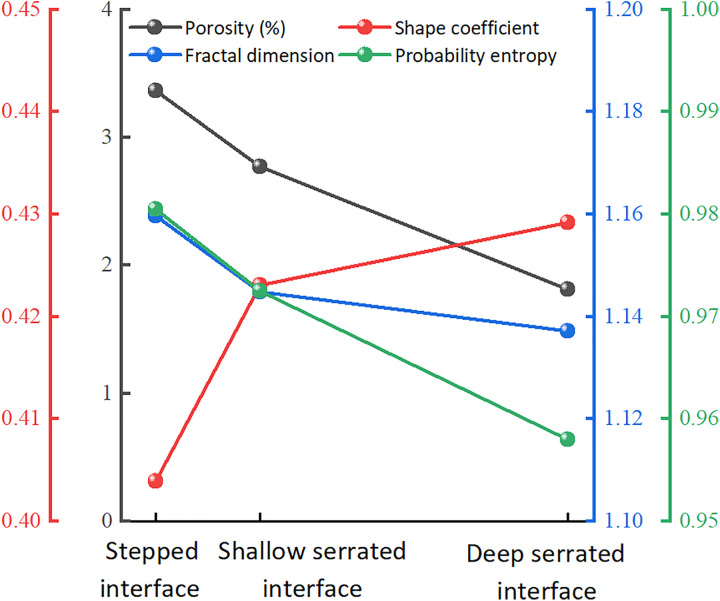
The change of microstructure parameters under different contact interface morphology.

The trends of relevant microstructural parameters are shown in Fig 15. As the contact interface changes from stepped to deep serrated (indicating increased interface roughness), the number of pores and average pore area decrease, leading to a reduction in porosity. This is due to the increased roughness replacing larger pores with smaller and micro-pores, resulting in a tighter arrangement of particles and enhanced interlocking, thereby increasing shear strength. The average shape coefficient of the pores rises with an increased interface roughness, indicating a reduction in larger pores and an increase in smaller pores, trending toward greater isotropic stability. In contrast, the average fractal dimension decreases, suggesting that the pore structure is less developed, more dispersed, and enhanced in stability. Furthermore, both pore probability entropy and the directional fractal dimension decrease as interface roughness increases, reflecting a more pronounced directional alignment of particles along the shear displacement and the surfaces of the protruding bodies, resulting in a more orderly pore distribution. This observation is consistent with the failure modes (Fig 9). Samples from the deep and shallow serrated interfaces primarily exhibit sliding along the interface with localized shear failure, while the stepped interface demonstrates direct shear failure.

**Fig 16:**
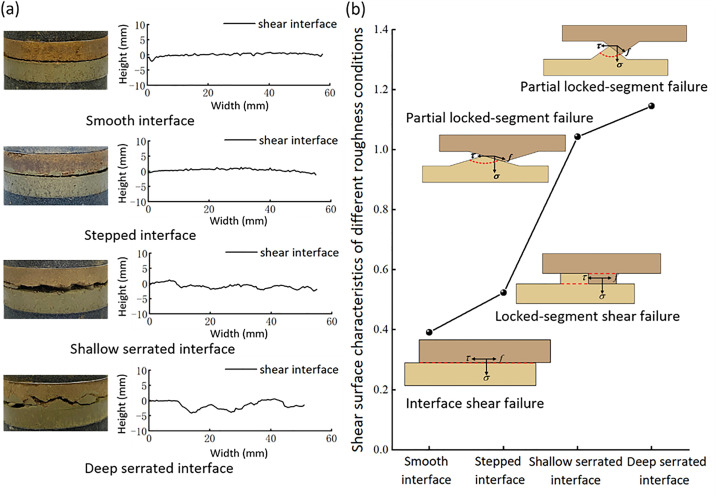
Macroscopic shear failure surface characteristics and shear failure modes of specimens with different contact interface morphologies. (a) Macroscopic shear failure surface characteristics of samples with different contact interface; (b) Roughness and shear failure mode of macroscopic shear failure surface with different contact interface morphology.

A standardized reference line is established on the side view of the specimen after failure. The contour of the shear failure surface is extracted by using the image digitization tool, and the roughness characteristics of the shear surface with different interface morphology are analyzed (Fig 16a). The roughness was quantified by calculating the deviation between the shear failure curve and the reference line. The calculated roughness values for smooth, stepped, shallow serrated, and deep serrated failure interfaces were 0.3914, 0.5239, 1.0434, and 1.145, respectively. Analysis of the shear surface morphology of different interface types revealed that the macroscopic shear failure patterns of loess-mudstone samples could be categorised into three distinct modes (Fig 16b): (I) Interface shear failure: In smooth interface specimens, shear failure primarily occurred as sliding along a flat interface. The upper loess layer moved along the contact surface, resulting in a smooth failure plane with low roughness [57]. (II) Locked-segment shear failure: In stepped interface specimens, failure was characterised by the direct shearing of the locked segments. During the shearing process, the locked segments were entirely severed at their base, resulting in a rougher failure surface compared to smooth interface specimens. (III) Partial locked-segment failure: In shallow and deep serrated interface specimens, the shear process exhibited an upslope climbing effect along the leading face of the triangular locked segments, while voids formed on the trailing face. After reaching a certain height, part of the locked segments was sheared off [58]. Due to differences in undulation angles, the failure positions in shallow and deep serrated interfaces varied [34]. In deep serrated interfaces, shearing occurred closer to the base of the locked segments, leading to a rougher shear surface.

### 4.3. Disaster effect of loess-mudstone interface

**Fig 17:**
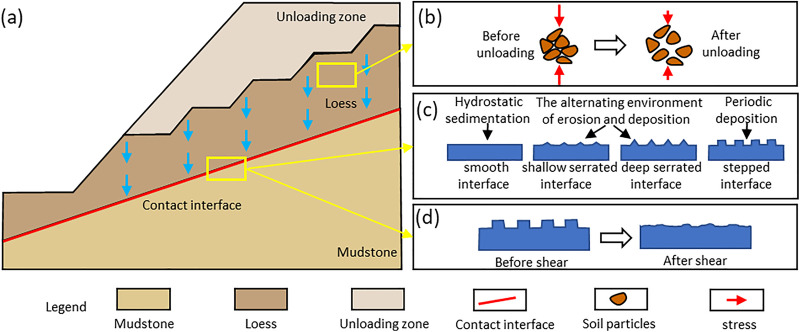
Principle diagram of disaster effect of loess-mudstone landslide: (a) Schematic diagram of loess-mudstone slope; (b) The change of dry density of overlying loess before and after unloading; (c) Different sedimentary environments form different interface morphology; (d) Interface roughness decreases after shearing process.

The large-scale slope excavation and urban construction projects in the Loess Plateau of China have formed many steep slopes with loess-mudstone interface similar to Fig 17a [59]. However, current stability assessments for these loess–mudstone slopes, which are prone to landslides, often adopt a conservative approach by using the strength parameters of loess as the lower bound [60,61]. According to the experimental results of this study, the interface strength is significantly lower than that of loess, indicating that stability assessments for these potentially unstable loess–mudstone slopes should fully incorporate the shear strength parameters of the interface composite. Additionally, in stability assessments under adverse conditions, it is crucial to consider the interface’s strength response to moisture, dry density, and roughness, as the weakening of interface strength under varying moisture and dry density conditions is significantly greater than that of loess alone. On the one hand, assessments should focus on the permeability differences between loess and mudstone [62,63], as these differences determine water accumulation at the interface and the formation of low friction band (Fig 13a). So, impermeability measures at the interface are particularly important in the stabilisation of loess–mudstone slopes. On the other hand, the consolidation conditions of the overlying soil (formation age) should also be evaluated, as loess with a shorter consolidation time generally exhibits lower dry density characteristics [64]. Furthermore, during the hillside excavation in city-building process, certain slope levelling measures, such as the slope release method commonly used on the Loess Plateau, can lead to unloading rebound effects, thereby altering the dry density characteristics of the overlying soil at the interface (Fig 17b). This aspect is often overlooked in the stability assessment of loess–mudstone slopes that have undergone slope release. In addition, the sedimentary environment of the loess–mudstone interface should be considered, as it significantly alters the contact properties of the interface [65,66], thereby affecting interface roughness and ultimately controlling shear strength (Fig 17c). For the risk assessment of loess-mudstone landslides that have occurred, the disaster effect should be considered that the landslide shear process significantly weakens the interface roughness [67], thereby reducing the interface shear strength (Fig 17d).

## 5. Conclusions

In this study, the loess-mudstone contact interface was taken as the research object, and the combination of indoor data collection, direct shear test and SEM analysis was used. The effects of moisture content, dry density and interface morphology on shear strength were studied by combining experimental data with microstructure observation. The study clarifies the macroscopic mechanical properties of loess-mudstone interface under different influencing factors, and reveals the microscopic mechanism of controlling the change of shear strength.

(1) Under different water content and dry density conditions of the upper loess, the shear strength of the smooth contact surface is always lower than that of homogeneous loess and mudstone. The smooth contact surface is the weak area of the loess-mudstone double-layer heterogeneous structure. The fundamental reason is that the dense and low permeability of the underlying mudstone leads to the accumulation of water at the interface, which makes the adjacent soil enter the over-wet soft plastic state and significantly reduces the shear strength.
(2) The moisture content and dry density of the upper loess are important factors affecting the shear strength of the loess-mudstone interface. Under the same dry density condition, the increase of water content leads to the decrease of shear strength at the contact surface. Moisture acts as a lubricant, softens clay cements, and weakens friction and occlusion between particles. On the contrary, at the same moisture content, the increase of dry density improves the shear strength, which is due to the change of particle arrangement from loose to tight, and the enhanced inter-particle occlusion.
(3) The interface morphology also has a significant effect on the shear strength. Under the same normal stress conditions, the shear strength of the contact surface is ranked as follows: deep serrated > shallow serrated > stepped > smooth. When the roughness of the contact surface is reduced from deep jagged to smooth, the cohesion is reduced by 42% and the friction angle is reduced by 13%. It shows that the change of interface morphology mainly affects the strength by affecting the cohesion, and the enhancement effect decreases with the increase of normal stress. Based on the shear surface morphology of different interface types, the macroscopic shear failure modes of loess-mudstone samples can be classified into three categories: Interface shear failure (smooth interfaces), Locked-segment shear failure (stepped interfaces), and Partial locked-segment failure (shallow and deep serrated interfaces).
(4) In the risk assessment of loess–mudstone slopes, the shear strength parameters of the interface composite should be fully considered. Additionally, in assessments under adverse conditions, it is essential to account for the interface’s strength response to moisture, dry density, and roughness. On the one hand, attention should be given to the permeability differences between loess and mudstone, as these differences determine water accumulation at the interface and the formation of low friction band. On the other hand, the consolidation conditions of the overlying soil and the unloading rebound effects caused by engineering excavation should be evaluated. In addition, for the risk assessment of loess mudstone landslides that have occurred, it should be considered that the disaster effect caused by shear process significantly weakens the interface roughness, thereby reducing the interface shear strength.

By analyzing the macroscopic mechanical properties of the loess-mudstone contact interface under different influencing factors, it provides an important reference for the risk assessment and prevention of such slopes. Our research results emphasize the influence of the three main factors of moisture content, dry density and interface morphology of the upper loess on the mechanical properties of the loess-mudstone interface, which is helpful to understand the influencing factors and internal mechanism of the interface strength change. However, the artificial preparation interface in the test is a simplification of the natural complex interface. The actual interface may have mineral weathering film, mudded interlayer or irregular fluctuation, and its influence still needs further study. This requires more relevant data of the actual slope interface and new complex interface preparation methods. Relevant research work is continuing to advance.

## Supporting information

S1 Fig Shear stress–shear displacement relationships for pure loess at different moisture contents, the loess–mudstone smooth interface under various upper loess moisture contents, and the control group mudstone: loess at (a) 10%, (b) 13%, (c) 16%, and (d) 19% moisture contents; (e) smooth interface with upper loess at 10%, (f) 13%, (g) 16%, and (h) 19% moisture contents; (i) control group mudstone. (PNG)

S2 Fig Shear stress–shear displacement relationships for pure loess at different dry densities, the loess–mudstone smooth interface under various upper loess dry densities, and the control group mudstone: loess with a dry densities of (a) 1.4, (b) 1.45, (c) 1.5, and (d) 1.55 g/cm^3^; smooth interface with upper loess at dry densities of (e) 1.4; (f) 1.45; (g) 1.5; and (h) 1.55 g/cm^3^; (i) control group mudstone. (PNG)

S3 Fig Shear stress–shear displacement relationship of loess–mudstone contact interface in different forms: (a) smooth; (b) stepped; (c) shallow serrated; (d) deep serrated. (PNG)
