# Supplementary figures and images for: Study on the influencing factors of shear strength of loess mudstone interface
Source: PLoS One. 2026 Feb 3;21(2):e0342023. doi: 10.1371/journal.pone.0342023 (PMC12867217; doi:10.1371/journal.pone.0342023)

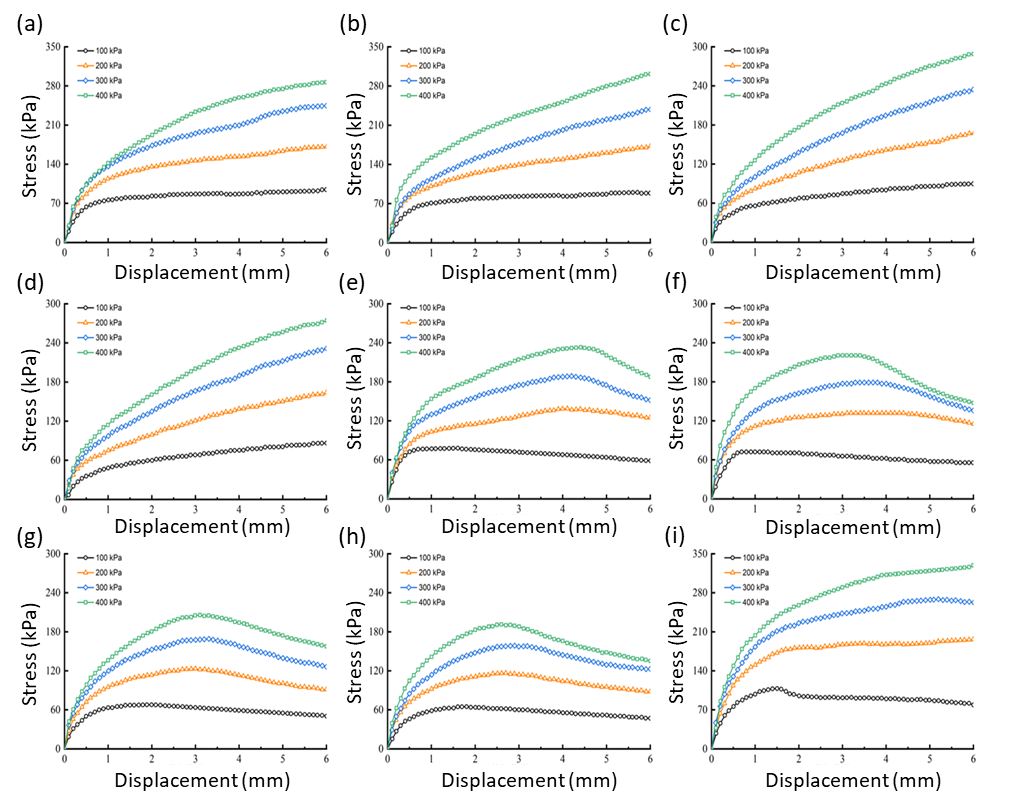

Supplement: S1 Fig — (PNG) [file pone.0342023.s001.png]

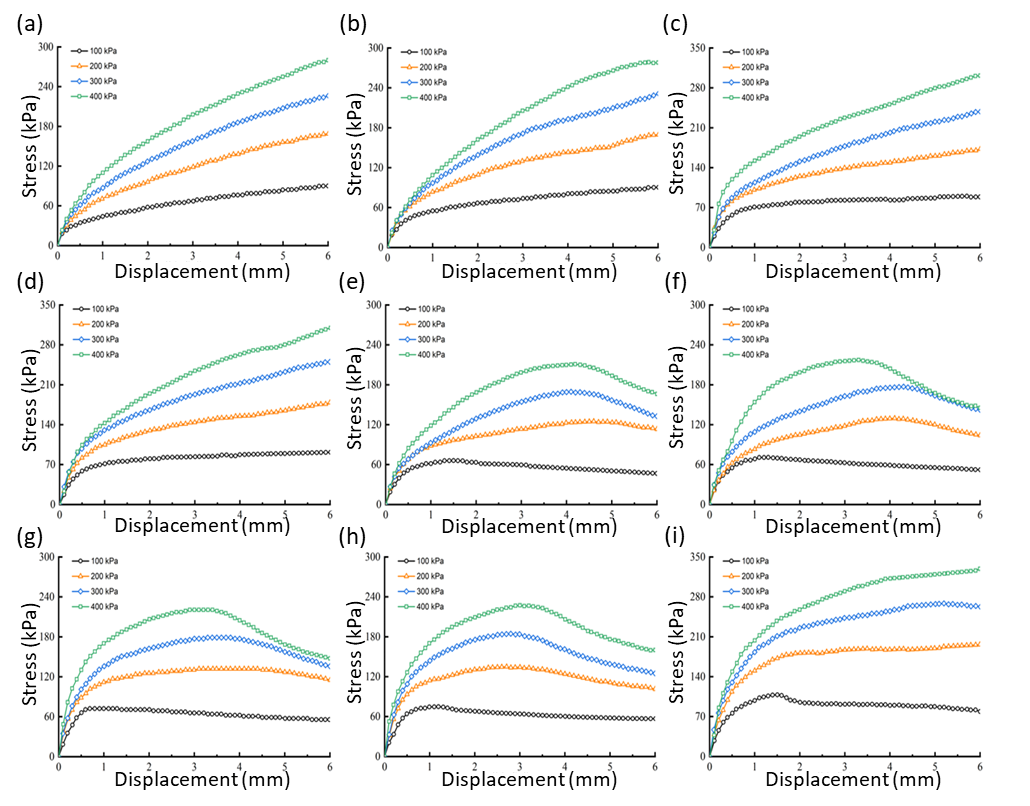

Supplement: S2 Fig — (PNG) [file pone.0342023.s002.png]

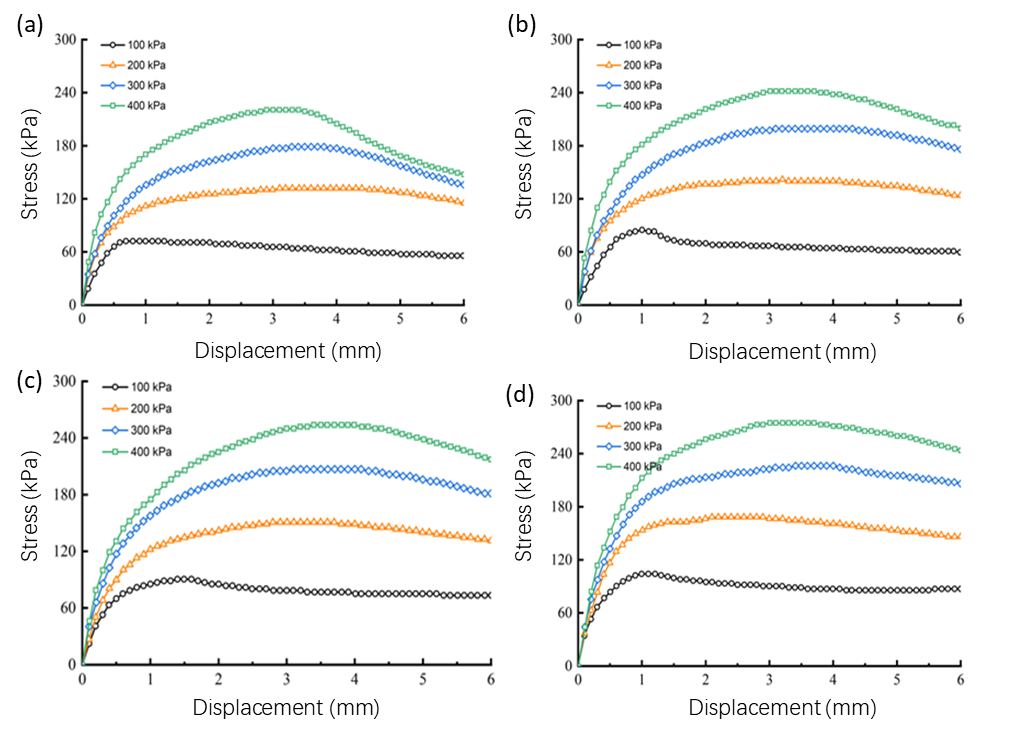

Supplement: S3 Fig — (PNG) [file pone.0342023.s003.png]
